# Data on cardiac defects, morbidity and mortality in patients affected by RASopathies. CARNET study results

**DOI:** 10.1016/j.dib.2017.11.085

**Published:** 2017-12-02

**Authors:** Giulio Calcagni, Giuseppe Limongelli, Angelo D'Ambrosio, Francesco Gesualdo, Maria Cristina Digilio, Anwar Baban, Sonia B. Albanese, Paolo Versacci, Enrica De Luca, Giovanni B. Ferrero, Giuseppina Baldassarre, Gabriella Agnoletti, Elena Banaudi, Jan Marek, Juan P. Kaski, Giulia Tuo, Maria Giovanna Russo, Giuseppe Pacileo, Ornella Milanesi, Daniela Messina, Maurizio Marasini, Francesca Cairello, Roberto Formigari, Maurizio Brighenti, Bruno Dallapiccola, Marco Tartaglia, Bruno Marino

**Affiliations:** a Department of Pediatric Cardiology and Cardiac Surgery, Bambino Gesù Children's Hospital, IRCCS, Rome, Italy; b Cardiologia SUN, Monaldi Hospital, II University of Naples, Naples, Italy; c Multifactorial Disease and Complex Phenotype Research Division, Bambino Gesù Children's Hospital, IRCCS, Rome, Italy; d Genetics and Rare Diseases Research Division, Bambino Gesù Children's Hospital, IRCCS, Rome, Italy; e Pediatric Cardiology, Department of Pediatrics, Sapienza University, Rome, Italy; f Department of Pediatric and Public Health Sciences, Città della Salute e della Scienza, University of Turin, Italy; g Cardiorespiratory Unit, Great Ormond Street Hospital for Children, London, UK; h Centre for Inherited Cardiovascular Diseases, Great Ormond Street Hospital, London, UK; i Department of Woman and Child's Health, Pediatric Cardiology, University of Padova, Padua, Italy; j Cardiovascular Department, Giannina Gaslini Institute, Genoa, Italy; k Cardiology and Cardiac Surgery, Sant’Orsola Malpighi Hospital, Bologna, Italy; l UCL Institute of Cardiovascular Science, London, UK

## Abstract

A comprehensive description of morbidity and mortality in patients affected by mutations in genes encoding for signal transducers of the RAS-MAPK cascade (RASopathies) was performed in our study recently published in the International Journal of Cardiology. Seven European cardiac centres participating to the CArdiac Rasopathy NETwork (CARNET), collaborated in this multicentric, observational, retrospective data analysis and collection. In this study, clinical records of 371 patients with confirmed molecular diagnosis of RASopathy were reviewed. Cardiac defects, crude mortality, survival rate of patients with 1) hypertrophic cardiomyopathy (HCM) and age <2 years or young adults; 2) individuals with Noonan syndrome and pulmonary stenosis carrying *PTPN11* mutations; 3) biventricular obstruction and *PTPN11* mutations; 4) Costello syndrome or cardiofaciocutaneous syndrome were analysed. Mortality was described as crude mortality, cumulative survival and restricted estimated mean survival. In particular, with this Data In Brief (DIB) paper, the authors aim to report specific statistic highlights of the multivariable regression analysis that was used to assess the impact of mutated genes on number of interventions and overall prognosis.

**Specifications Table**

**Table:** 

Subject area	*Clinical Cardiology, Genetics, Rare Diseases, Morbility and Mortality*
More specific subject area	*RASopathies, congenital heart defects, Hypertrophic Cardiomyopathy, Costello Syndrome, Noonan Syndrome, LEOPARD syndrome, cardiofaciocutaenous Syndrome*
Type of data	*Table, figures*
How data was acquired	*Clinicians analysis*
Data format	*Filtered and analyzed*
Experimental factors	*Molecular diagnosis was performed through a combination of Sanger sequencing and targeted resequencing directed to scan the entire coding sequence of CBL, PTPN11, SOS1, KRAS, HRAS, NRAS, SHOC2, RAF1, BRAF, MAP2K1 and MAP2K2* genes.
Experimental features	*Analysis were retrospectively performed.*
	*Tables report the correlation between genetic testing and outcomes of the related research article.*
	*No experimental features were used or applied to data collection and analysis.*
Data source location	*Rome, Bologna, Padua, Turin, Genoa, Naples (Italy)*
	*London (United Kingdom)*
Data accessibility	*Data is with this article and not in a public repository*
Related research article	*Cardiac defects, morbidity and mortality in patients affected by RASopathies. CARNET study results.*
	*Calcagni G, Limongelli G, D'Ambrosio A, Gesualdo F, Digilio MC, Baban A, Albanese SB, Versacci P, De Luca E, Ferrero GB, Baldassarre G, Agnoletti G, Banaudi E, Marek J, Kaski JP, Tuo G, Russo MG, Pacileo G, Milanesi O, Messina D, Marasini M, Cairello F, Formigari R, Brighenti M, Dallapiccola B, Tartaglia M, Marino B.*
	*Int J Cardiol. 2017 Oct 15;245:92-98.*doi:10.1016/j.ijcard.2017.07.068. Epub 2017 Jul 21.

**Value of the data**

• Rasopathy syndromes are a well known entity and specific genotype-cardiac phenotype correlation have been established. Limited information is available on genotype, phenotype and clinical/surgical cardiac outcomes in these patients, thus more data need to be collected and analysed.
• The large cohort recently published in the related research article provides specific information on cardiac morbidity and mortality about RASopathies. In this study the authors demonstrated that cardiac mortality is relatively uncommon in these disorders and that in specific subsets of patients, risk factors for surgical mortality or sudden death can be observed.
• Statistical correlations reported in tables and figures of the present DIB paper focus in details on genes description for each syndrome, genetic testing, mortality rate and surgical re-intervention.
• Defining the worse cardiac phenotype spectrum will be essential to target specific treatment phenotype/gene/mutation-specific in patient affected with RASopathies. This knowledge also allow to optimize the counselling for family and for patients affected by these genetic disorders.

## 1 Data

Data shared consist in peculiar details and explanation of methods and techniques of the statistical analysis of patients’ reports and overall data. Moreover, we have correlated patients and parameters in the following figures and tables.

**Fig. 1:**
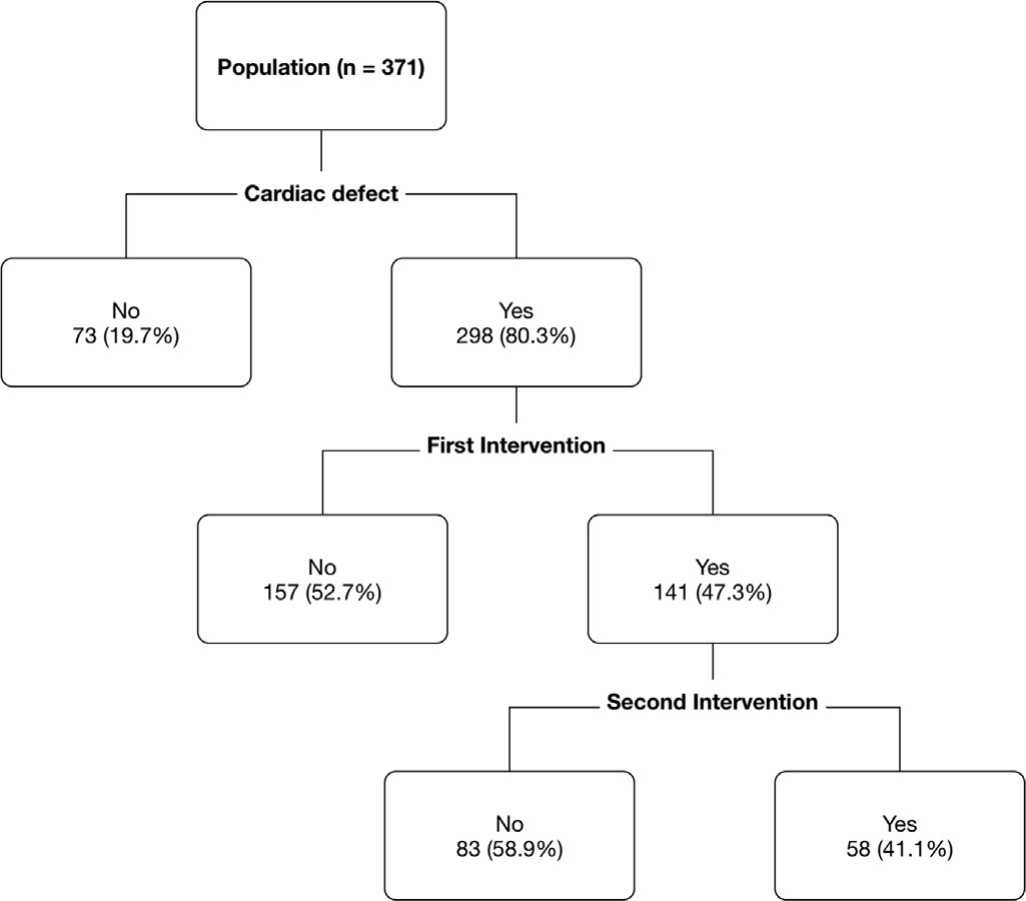
Patient's characteristics and procedures.

– Fig. 1: Patients characteristics and procedures are described in details in DIB, which is a detailed analysis of the first part of Table 1 of the related research article;

**Table 1:** Distribution of the mutated disease genes by clinical diagnosis.

Gene (N,%)	NS	NSML	CFCS	CS	Total
*PTPN11*	216	42	0	0	258
*SOS1*	44	0	0	0	44
*BRAF*	6	1	15	0	22
*RAF1*	17	2	0	0	19
*SHOC2*	9	0	0	0	9
*HRAS*	1	0	0	7	8
*MAP2K1*	0	0	5	0	5
*MAP2K2*	0	0	2	0	2
*KRAS*	2	0	0	0	2
*CBL*	1	0	0	0	1
*NRAS*	1	0	0	0	1
Total	297	45	22	7	371
– Table 1 shows the distribution of mutated disease genes by syndrome;
– In Fig. 2, multiple forest plots reports effect sizes and BCa 95% Cls of the adjusted regression analyses. This figure refers to the re-intervention of patients, and to peculiar aspects of Fig. 1 of the related research article;

**Fig. 2:**
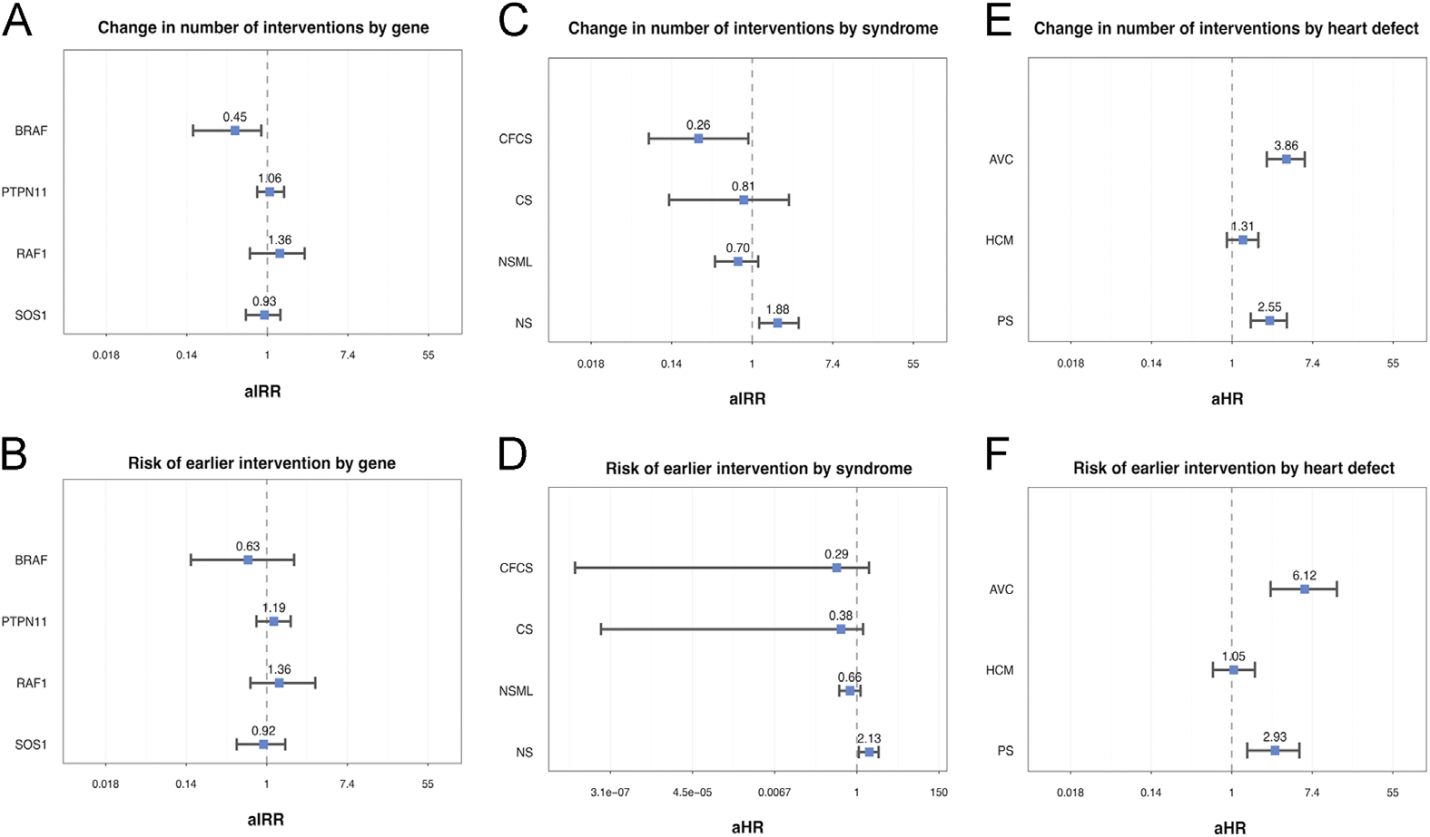
Forest plots reporting effect, size and BCa 95% Cls of the adjusted regression analysis.
– Additional supplementary tables: the relation between morbility and genetic mutation, syndromes and cardiac defects is reported. Data were presented as median and range, mean±standard deviation (SD), or percentages and CI, as appropriate. These supplementary tables better explain some correlations observed in Fig. 1 of related research article.

## 2 Experimental design, materials and methods

The cardiac defects that were taken into account in the analyses were selected based on the clinical impact of each single defect on the natural history of disease [1]. HCM, PS, and AVC were selected as principal cardiac defects. Although ASD, VSD and valve anomalies were relatively frequent in the present study cohort, they were excluded from the statistical analysis due to poor clinical impact (i.e. ASD) and/or to their frequent association with other major cardiac defects (e.g., mitral valve anomalies and HCM). Therefore, although the frequency of every cardiac defects are described, only HCM, PS, and AVC were investigated more thoroughly.

We investigated all relevant gene mutations present in our population, but given the rarity of most genetic defects in RASophaties, we decided to focus on mutations of BRAF, PTPN11, RAF1 and SOS1. Especially, when plotting the results of the analyses, we reported only the results relative to this subset of genes in order to increase clarity and interpretability.

We performed a number of inferential analyses using multivariable regression models.

The models were chosen according to the type of dependent variable investigated.

Poisson regression was used to study the association between mutated genes and the number of procedures received by each patient with a cardiac defect (interpreted as a count variable), adjusting for sex, presence of PS, HCM, AVC or OHD, and natural logarithm of years of follow-up, to take into account the length of the follow up for each subject. Through Poisson regression, we investigated the association of each syndrome with the number of interventions, adjusting for sex, type of heart defect and length of follow up (transformed as above), and association between presence of PS, HCM, AVC and OHD, and the number of interventions, correcting for sex, gene, and length of follow up (transformed as above).

Regression analyses were performed using a Bayesian regularization approach with a non-informative prior with a Cauchy distribution, which allows for more stable estimates in case of unbalanced class distribution or perfect/quasi-perfect predictor-outcome separation, avoiding the production of extreme estimates which are probable to be random fluctuation, therefore improving reproducibility of results [2].

Kaplan-Meier (KM) curves were used to describe the incidental risk of intervention and mortality, stratified by time, for each mutated gene, syndrome and heart defect; 95% confidence intervals (95% CI) are shown for the cumulative survival KM plots. In the population of subjects with heart defects, Cox proportional hazards models were used to assess the effect of having a mutated gene, a syndrome or a specific heart defect on the risk of intervention, respectively adjusting for sex and heart defect, sex and heart defect, sex and gene.

Variability of the estimates was assessed by applying a non-parametric bootstrap analysis with 5000 repetitions in order to be more robust in presence of non-normal distributions of the investigated effects, which is a common situation when facing complex, multifactorial phenomena [3].

Standardized effect sizes (sES), defined as the ratio between mean and SD of the bootstrap distributions of the predictors, were reported to assess the inferential strength of each predictor on the outcomes (the further from zero is the absolute value, the stronger is the effect).

Accuracy of the estimates is reported as bias-corrected and accelerated bootstrap 95% Confidence Intervals (BCa 95% CIs) [4]. BCa 95% CIs and effect sizes have been exponentiated in order to be interpreted as adjusted Odds Ratio (aOR) in case of logistic regressions, adjusted Incidence Rate Ratios (aIRR) in case of Poisson regressions, and adjusted hazard ratios (aHR) when considering the Cox model.

A relationship was considered significant when the BCa 95% CIs were either greater or smaller than the null effect. For each analysis, we took into account only subjects for whom the considered variables were present. The actual number of subjects that entered in each analysis is reported in each table.

We purposely decided not to report p-values since we believe this statistic can lead to a superficial if not mislead interpretation of research results [5], [6]. We believe that confidence intervals and effect sizes report all the information necessary for the interpretation of regression results, since they provide both a measure of uncertainty (the length of the confidence interval) and a estimation of the strength of the relationships (the effect sizes) between variables.

We also report the sES in order to provide a further synthetic statistic which join precision and strength of an effect, giving an approximate degree of inferential importance without misleading probabilistic implications. Many share our criticism of p value, therefore we decided to avoid using it in this publication.
