# Supplementary material for: Data on cardiac defects, morbidity and mortality in patients affected by RASopathies. CARNET study results
Source: Data Brief. 2017 Dec 2;16:649–54. doi: 10.1016/j.dib.2017.11.085 (PMC5847490; doi:10.1016/j.dib.2017.11.085)
Supplement: Supplementary file 2 — Supplementary material [file mmc2.pdf]

Supplemental Table A

Relationship between specific gene mutations and morbidity (number of interventions).

Mutated Gene (a)	aIRR (b)	Std Effect Size (c)	BCa 95% CIs (d)
BRAF	0.448	-1.82	0.158, 0.859
CBL	0.373	-3.99	0.191, 0.466
HRAS	0.811	-0.604	0.132, 2.11
KRAS	6.57	10.7	4.59, 9.16
MAP2K1	0.189	-3.94	0.0687, 0.349
NRAS	0.301	-4.43	0.14, 0.351
PTPN11	1.06	0.363	0.777, 1.51
RAF1	1.36	0.83	0.648, 2.52
SHOC2	1.42	0.228	0.278, 3.91
SOS1	0.931	-0.384	0.586, 1.38

(a) Comparison of patients having the specified gene mutation versus patients having a mutation in the other genes.

(b) IRR are adjusted for sex of the patient, for cardiac defect and for the log of years of observation.

(c) Standardized effect size is calculated as mean over standard deviation of the bootstrap distribution.

(d) Bootstrap bias-corrected accelerated confidence intervals

Supplemental Table B

Cox analysis between gene mutations and risk of intervention during the observation period.

Mutated Gene (a)	aHR (b)	Std Effect Size (c)	BCa 95% CIs (d)
BRAF	0.628	-0.374	0.152, 1.97
CBL	1.15 x 10^6	-24.5	7.92 x 10^-7, 2.61 x 10^-6
HRAS	0.381	-0.835	1.73 x 10^-7, 1.42
KRAS	4.43	6.45	2.76, 7.03
MAP2K1	1.72 x 10^7	-21.7	5.26 x 10^-8, 6.28 x 10^-7
NRAS	4.80 x 10^7	-25.2	2.97 x 10^-7, 7.62 x 10^-7
PTPN11	1.19	0.891	0.773, 1.81
RAF1	1.36	0.595	0.666, 3.33
SHOC2	1.32	-0.143	2.02 x 10^-7, 6.86
SOS1	0.924	-0.264	0.474, 1.58

(a) Comparison of patients having the specified gene mutation versus patients having a mutation in the other genes.
(b) HR are adjusted for sex of the patient and for cardiac defect.
(c) Standardized effect size is calculated as mean over standard deviation of the bootstrap distribution.
(d) Bootstrap bias-corrected accelerated confidence intervals.

Supplemental Table C

Relationship between syndromes and morbidity (number of interventions).

Syndrome (a)	aIRR (b)	Std Effect Size (c)	BCa 95% CIs (d)
CFCS	0.264	-2.14	0.0767, 0.909
CS	0.811	-0.606	0.127, 2.49
NSML	0.704	-1.26	0.397, 1.16
NS	1.88	2.46	1.19, 3.17

(a) Comparison of patient with the syndrome versus patients without it.

(b) IRR are adjusted for sex of the patient, for cardiac defect and for the log of years of observation.

(c) Standardized effect size is calculated as mean over standard deviation of the bootstrap distribution.

(d) Bootstrap bias-corrected accelerated confidence intervals

Supplemental Table D

Cox analysis between syndromes and risk of intervention during the observation period.

Syndrome (a)	aHR (b)	Std Effect Size (c)	BCa 95% CIs (d)
CFCS	0.294	-0.606	3.56 x 10^-8, 2.09
CS	0.381	-0.833	1.73 x 10^-7, 1.46
NSML	0.658	-1.42	0.343, 1.25
NS	2.13	2.62	1.12, 3.75

(a) Comparison of patient with the syndrome versus patients without it.

(b) HR are adjusted for sex of the patient and for cardiac defect.

(c) Standardized effect size is calculated as mean over standard deviation of the bootstrap distribution.

(d) Bootstrap bias-corrected accelerated confidence intervals.

Supplemental Table E

Relationship between cardiac defects and morbidity (number of interventions).

Heart Defect (a)	aIRR (b)	Std Effect Size (c)	BCa 95% CIs (d)
AVC	3.86	5.66	2.37, 6.08
HCM	1.31	1.23	0.88, 1.92
PS	2.55	4.2	1.59, 3.9

(a) Comparison of patient with specified heart defect versus patients without it.

(b) IRR are adjusted for sex of the patient, for mutated genes, for cardiac defect and for the log of years of observation.

(c) Standardized effect size is calculated as mean over standard deviation of the bootstrap distribution.

(d) Bootstrap bias-corrected accelerated confidence intervals

Supplemental Table F

Cox analysis between cardiac defects and risk of intervention during the observation period.

Heart Defect (a)	aHR (b)	Std Effect Size (c)	BCa 95% CIs (d)
AVC	6.12	4.41	2.62, 13.6
HCM	1.05	0.0884	0.627, 1.78
PS	2.93	3.45	1.47, 5.36

(a) Comparison of patient with specified heart defect versus patients without it.

(b) HR are adjusted for sex of the patient, for cardiac defect and for mutated gene.

(c) Standardized effect size is calculated as mean over standard deviation of the bootstrap distribution.

(d) Bootstrap bias-corrected accelerated confidence intervals.
